# Keine abskopalen Effekte bei Kombination von Immuntherapie und stereotaktischer Radiotherapie bei Merkel-Zell-Karzinomen

**DOI:** 10.1007/s00066-023-02078-6

**Published:** 2023-04-12

**Authors:** Sören Schnellhardt, Udo S. Gaipl, Markus Hecht

**Affiliations:** 1 Klinik für Strahlentherapie und Radioonkologie, Universitätsklinikum des Saarlandes, Kirrberger Straße, Gebäude 6.5, 66421 Homburg, Deutschland; 2 Translational Radiobiology, Strahlenklinik, Universitätsklinikum Erlangen, Friedrich-Alexander-Universität Erlangen-Nürnberg (FAU), 91054 Erlangen, Deutschland

## Hintergrund und Ziele

Die Therapie des lokal fortgeschrittenen und metastasierten Merkel-Zell-Karzinoms wurde in den vergangenen Jahren durch die Einführung von Immuncheckpointinhibitoren (ICI) revolutioniert. Während herkömmliche Chemotherapeutika bei oft schwerwiegenden Nebenwirkungen in der Regel nur zu kurzfristigem Erfolg führten, konnte durch den Einsatz von ICI in Studien bei über einem Drittel der Patienten ein zum Teil langfristiges Therapieansprechen erreicht werden. In Deutschland ist in der fernmetastasierten Situation die Monotherapie mit dem PD-L1-Antikörper Avelumab zugelassen, in den USA wird auch Pembrolizumab (Zielstruktur PD-1) eingesetzt. Systemtherapieoptionen bei einem Progress unter ICI-Therapie sind jedoch gering und die Prognose schlecht. Die Effektivität einer doppelten Immuncheckpointblockade, wie sie beispielsweise in der Therapie des Nierenzellkarzinoms mit Ipilimumab und Nivolumab eingesetzt wird, ist sowohl in der progredienten Krankheitssituation als auch bei ICI-naiven Patient:innen mit Merkel-Zell-Karzinom unklar. Auch die Hypothese einer möglichen Verbesserung des systemischen Therapieansprechens von ICI durch eine lokale Strahlentherapie im Rahmen des sogenannten systemischen, abskopalen Effekts ist in diesem Kontext offen.

In der hier vorliegenden zweiarmigen, nichtverblindeten, randomisierten Studie von Kim et al. wurden daher anhand von 50 Patienten mit lokal fortgeschrittenem oder fernmetastasiertem Merkel-Zell-Karzinom mehrere primäre Fragestellungen untersucht: Wie ist das Ansprechen auf eine doppelte Immuncheckpointblockade mit Ipilimumab (Zielstruktur CTLA-4) und Nivolumab (Zielstruktur PD-1)? Verbessert die stereotaktische Bestrahlung (SBRT) einer Läsion den systemischen Therapieerfolg? Und profitieren auch Patienten, welche bereits mit einem PD-1- oder PD-L1-Inhibitor vorbehandelt wurden, von einer Therapie mit Ipilimumab und Nivolumab?

## Patienten und Methoden

In die zweiarmige, nichtverblindete Phase-II-Studie konnten Patientinnen und Patienten mit nichtresezierbarem, rezidiviertem oder metastasiertem Merkel-Zell-Karzinom mit mindestens zwei histologisch gesicherten bildmorphologisch oder klinisch messbaren Tumorläsionen eingeschlossen werden. Eine Vorbehandlung mittels Chemo- oder Immuntherapie war bei progredienter Erkrankung zugelassen, eine Vorbestrahlung nur an nicht im Zuge der Studie zu messenden Läsionen.

Die Behandlung erfolgte in zwei Zentren in den USA. Die Patienten wurden nach Vortherapie mit ICI stratifiziert in zwei Gruppen randomisiert und mit Nivolumab (240 mg Absolutdosis, q14d) und Ipilimumab (1 mg/kg Körpergewicht, q42d) behandelt (Therapiearm A). Im Therapiearm B wurde während der zweiten Therapiewoche zusätzlich eine stereotaktische Bestrahlung (SBRT) mindestens einer Läsion mit 24 Gy in 3 Fraktionen durchgeführt. Mindestens eine messbare Läsion musste unbestrahlt bleiben. Primärer Endpunkt war die objektive Ansprechrate (ORR), definiert als Anteil der Patienten, welche ein komplettes oder partielles Krankheitsansprechen nach „immune-related response evaluation criteria in solid tumours“ (irRECIST) aufwiesen. Sekundäre Endpunkte waren das progressionsfreie Überleben (PFS), Gesamtüberleben (OS) und die lokale Kontrolle (LC) bestrahlter Läsionen.

## Ergebnisse

Zwischen März 2017 und Dezember 2021 konnten 50 Patienten in die Studie eingeschlossen werden. 76 % wiesen eine Erkrankung im Stadium IV auf. Es gab keine statistisch signifikanten Unterschiede bezüglich der Verteilung klinischer Charakteristika zwischen den beiden Therapiearmen. In Therapiearm A (Ipilimumab/Nivolumab) waren 12 Patienten mit ICI vortherapiert, in Therapiearm B (Ipilimumab/Nivolumab + SBRT) waren es 14.

Die ORR betrug 72 % in Therapiearm A und 52 % in Therapiearm B. Dieser Unterschied war nicht statistisch signifikant (*p* = 0,26). Auch bezüglich PFS und OS ergaben sich keine Unterschiede zwischen den Therapiearmen. Die Analyse nach ICI-Vorbehandlungsstatus ergab eine 100 % ORR bei ICI-naiven Patienten (41 % mit komplettem Ansprechen), während sie bei ICI-vortherapierten Patienten 31 % (15 % mit komplettem Ansprechen) betrug. Höhergradige therapiebedingte Nebenwirkungen traten bei 10 Patienten in Therapiearm A (40 %) und bei 8 Patienten in Therapiearm B (32 %) auf.

## Schlussfolgerung der Autoren

Die Kombination Ipilimumab/Nivolumab ist beim rezidivierenden oder metastasierenden Merkel-Zell-Karzinom eine wirksame Therapieform mit einem akzeptablen Sicherheitsprofil und könnte nach weiterer Bestätigung der Ergebnisse sowohl als Erstlinien- als auch als Salvage-Therapie zum Einsatz kommen. SBRT führte zu keiner Verbesserung der Ansprechrate.

## Kommentar

Das fortgeschrittene und fernmetastasierte Merkel-Zell-Karzinom gilt als aggressive und chemotherapeutisch schwierig zu behandelnde Tumorerkrankung. Seit 2017 ist in Deutschland Avelumab in der metastasierten Situation zugelassen. In einer Phase-II-Studie konnte damit in der Erstlinientherapie eine Ansprechrate von 62 % erreicht werden [1].

In der hier vorliegenden Studie führte die doppelte Immuncheckpointblockade mit Ipilimumab/Nivolumab bei ICI-naiven Patienten zu einer Ansprechrate von beeindruckenden 100 %. Aber auch in der ICI-vortherapierten Situation konnte eine Ansprechrate von 31 % festgestellt werden. Als eine mögliche Erklärung für dieses überdurchschnittlich gute Therapieansprechen des Merkel-Zell-Karzinoms auf ICI gilt die starke Expression von Neoantigenen bzw. viraler Antigene bei Polyomavirus-positiven Tumoren.

Aus strahlentherapeutischer Sicht ist in dieser Studie die Mituntersuchung des abskopalen Effekts besonders interessant. Der abskopale Effekt beschreibt das Konzept eines am ehesten immunvermittelten systemischen Ansprechens einer Tumorerkrankung auf eine Radiotherapie auch außerhalb des Bestrahlungsfelds. Dieses Phänomen wurde seit den 1950er-Jahren in verschiedenen präklinischen und klinischen Fallberichten und Studien beschrieben. Grob zusammengefasst geht man von einer systemischen tumorgerichteten Aktivierung des Immunsystems durch Präsentation von Tumorantigenen in einer immunstimulierenden Tumormikroumgebung nach einer Strahlentherapie aus. Diese Vorgänge sind jedoch, genau wie die Frage, warum dieser Effekt nicht bei jeder Strahlentherapie beobachtet wird, nicht abschließend geklärt [2].

Seit der klinischen Einführung von ICI wird in zahlreichen Studien untersucht, ob die systemische Wirkung von Immuntherapien durch den Einsatz von Strahlentherapie verbessert werden kann [3]. In der hier vorliegenden Studie von Kim et al. zum Merkel-Zell-Karzinom war dies nicht der Fall: Die Ansprechrate auf die doppelte Immuncheckpointblockade war im Therapiearm B (ICI + SBRT) mit 52 % sogar numerisch niedriger als im Therapiearm A (72 %, *p* = 0,26). Auch das mediane Gesamtüberleben (14,9 Monate vs. 9,7 Monate) und progressionsfreie Überleben (4,2 Monate vs. 2,7 Monate) bei ICI-vorbehandelten Patient:innen waren bei Hinzunahme von Strahlentherapie numerisch unterlegen. Bei ICI-naiven Patienten wurden das mediane OS und PFS nicht erreicht.

Dies ist zunächst als ein negatives Ergebnis für einen möglichen abskopalen Effekt in der ICI-Therapie von Merkel-Zell-Karzinomen zu werten, muss aber im Kontext von Studiendesign, Gruppengröße und auch des außergewöhnlich guten Ansprechens der ICI-naiven Gruppe bewertet werden. Die Autoren merken diesbezüglich an, dass ein möglicher prognostisch positiver Effekt der Bestrahlung bei einer Ansprechrate von 100 % bei ICI-naiven Patienten kaum zu erfassen wäre. Somit ist man zur Bewertung eines möglichen abskopalen Effekts auf die ICI-vortherapierte Gruppe angewiesen. Hier könnte jedoch das durch die vorangegangene Immuntherapie bereits stark modifizierte Tumormikromilieu das Auftreten eines abskopalen Effekts beeinflusst oder gar unterbunden haben.

Dem ist hinzuzufügen, dass die ICI-vortherapierte Gruppe lediglich 12 Patienten in Therapiearm A und 14 Patienten in Therapiearm B umfasste und somit einer nicht zu unterschätzenden statistischen Unsicherheit unterlag. Des Weiteren könnten Therapiemodalität und -sequenz ebenfalls Einfluss auf das Auftreten eines möglichen abskopalen Effekts haben. Beispielsweise war in einer Phase-II-Studie zur definitiven Radiochemotherapie von Kopf-Hals-Tumoren die sequenzielle PD-1-Inhibition einer simultanen Gabe, wie sie in dieser Studie durchgeführt wurde, überlegen [4].

Hinsichtlich der Dosierung weisen präklinische Daten darauf hin, dass im Vergleich zu 1 × 8 Gy bei 1 × 20 Gy deutlich weniger Immunstimulation auftritt und dass bei 3 × 8 Gy im Vergleich zu 2 × 8 Gy abskopale Antitumorimmunantworten, einhergehend mit einer verstärkten Expression gleich mehrerer suppressiver Immuncheckpointmoleküle, nicht mehr zu beobachten sind [5, 6]. Möglicherweise war die Dosis in der aktuellen Studie mit 3 × 8 Gy zu hoch gewählt, um in Summe immunstimulierende Effekte durch die Strahlentherapie zu induzieren.

Darüber hinaus ist bekannt, dass unterschiedliche Metastasen ein durch die Tumorheterogenität unterschiedliches Mikromilieu aufweisen. Daher ist es möglicherweise nicht ausreichend, die zur Bestrahlung geplante Metastase zufällig auszuwählen [7]. Eine Selektion immunologisch kritischer Metastasen könnte beispielweise mittels Radiomics erfolgen [3].

Diese bei Initiierung dieser Studie noch nicht bekannten Aspekte sollten in der Planung zukünftiger Studien berücksichtigt werden. Vor diesem Hintergrund bleibt das Ergebnis der deutschen Keynote-717/IMPORTANCE-Studie (NCT03386357) abzuwarten, in der eine fraktionierte Bestrahlung in Kombination mit Pembrolizumab bei Kopf-Hals-Tumoren erfolgt. Für den klinischen Alltag bleibt anzumerken, dass auch in Kombination mit ICI die Bestrahlung aller Metastasen bei Oligometastasierung der Bestrahlung nur einer einzelnen Läsion vorzuziehen ist [8].

## Fazit

Die doppelte Immuncheckpointblockade mit Ipilimumab/Nivolumab zeigte ein optimales Therapieansprechen bei ICI-naiven Patient:innen mit Merkel-Zell-Karzinomen und könnte auch nach ICI-Vortherapie eine Option sein. Die Hinzunahme einer stereotaktischen Strahlentherapie verbesserte das Ansprechen nicht. Allerdings weisen Studienergebnisse darauf hin, dass Therapiesequenz und -dosierung aus heutiger Sicht vermutlich nicht optimal gewählt wurden. Zudem erfolgte keine Selektion der zu bestrahlenden Metastasen. Das Resultat dieser Studie lässt daher nur eine Aussage über exakt dieses Therapiekonzept zu, jedoch nicht über immunmodulierende Effekte einer Strahlentherapie in Kombination mit ICI im Allgemeinen.

*S Schnellhardt, M. Hecht, Homburg/Saar*

*U.S. Gaipl, Erlangen*

## Interessenkonflikt

U.S. Gaipl: AstraZeneca (Advisory Boards, Forschungsfinanzierung); BMS (Advisory Board); Merck Serono (Vortragstätigkeit); MSD (Forschungsfinanzierung); Dr. Sennewald Medizintechnik (Advisory Board, Vortragstätigkeit). M. Hecht: Merck Serono (Advisory Boards, Vorträge, Reisemittel, Forschungsfinanzierung); MSD (Advisory Boards, Vorträge, Reisemittel, Forschungsfinanzierung); AstraZeneca (Advisory Boards, Vorträge, Reisemittel, Forschungsfinanzierung); Novartis (Forschungsfinanzierung); BMS (Advisory Boards, Vorträge, Reisemittel, Forschungsfinanzierung); Sanofi Genzyme (Advisory Boards, Forschungsfinanzierung); Teva (Reisemittel). S. Schnellhardt gibt an, dass kein Interessenkonflikt besteht.

## References

[1] D’Angelo SP, Russell J, Lebbe C (2018). Efficacy and safety of first-line avelumab treatment in patients with stage IV metastatic Merkel cell carcinoma: a preplanned interim analysis of a clinical trial. JAMA Oncol.

[2] Ngwa W, Irabor OC, Schoenfeld JD (2018). Using immunotherapy to boost the abscopal effect. Nat Rev Cancer.

[3] Sun R, Sundahl N, Hecht M (2020). Radiomics to predict outcomes and abscopal response of patients with cancer treated with immunotherapy combined with radiotherapy using a validated signature of CD8 cells. J Immunother Cancer.

[4] Clump DA, Zandberg DP, Skinner HD (2022). A randomized phase II study evaluating concurrent or sequential fixed-dose immune therapy in combination with cisplatin and intensity-modulated radiotherapy in intermediate- or high-risk, previously untreated, locally advanced head and neck cancer (LA SCCHN). J Clin Oncol.

[5] Vanpouille-Box C, Alard A, Aryankalayil MJ (2017). DNA exonuclease Trex1 regulates radiotherapy-induced tumour immunogenicity. Nat Commun.

[6] Ruckert M, Deloch L, Frey B (2021). Combinations of radiotherapy with vaccination and immune checkpoint inhibition differently affect primary and abscopal tumor growth and the tumor Microenvironment. Cancers.

[7] Brooks ED, Chang JY (2019). Time to abandon single-site irradiation for inducing abscopal effects. Nat Rev Clin Oncol.

[8] Schubert P, Rutzner S, Eckstein M (2020). Prospective Evaluation of All-lesion Versus Single-lesion Radiotherapy in Combination With PD-1/PD-L1 Immune Checkpoint Inhibitors. Front Oncol.

